# Formation and Clearance of NETs in Health and Disease

**DOI:** 10.3390/cells11244022

**Published:** 2022-12-12

**Authors:** Jasmin Knopf, Aparna Mahajan, Luis E. Muñoz, Martin Herrmann

**Affiliations:** 1 Department of Internal Medicine 3—Rheumatology and Immunology, Friedrich-Alexander University Erlangen-Nürnberg, Universitätsklinikum Erlangen, 91054 Erlangen, Germany; 2 Deutsches Zentrum für Immuntherapie (DZI), Friedrich-Alexander University Erlangen-Nürnberg and Universitätsklinikum Erlangen, 91054 Erlangen, Germany

Neutrophils are the most abundant innate immune cells in humans and the first line of defense against invading pathogens. Neutrophil extracellular trap (NET) formation was first described in 2004 as a mechanism for neutrophils to trap and kill bacteria [1]. Since then, NET formation has been described for many other pathogens and inert organic or inorganic materials. Currently, NET formation is considered not only beneficial for the host but also detrimental in many inflammatory and autoimmune conditions; a fine balance between NET formation and clearance is mandatory [2].

This Special Issue of *Cells* is dedicated to the current knowledge on NET formation concerning its clinical impact. To this end, the Special Issue contains two review articles. In the first article, Vitkov et al. entangle the role of disturbed NET formation and clearance in early onset vs. late-onset periodontitis [3], while Santocki and Kolaczkowska highlight what is known about the clearance of NETs and propose further strategies to study NET removal [4].

In their original research article, Schapher et al. demonstrated how the immune-mediated mechanism of NET formation promotes the development and growth of salivary stones, a common cause of obstructions in the salivary gland [5]. The authors also show that both calcium salt crystals and extracellular DNA from oral bacteria are potential inducers of NET formation, promoting sialolithogenesis and obstructive sialadenitis. 

The involvement of NETs and monocyte extracellular traps in drug crystal-related gastrointestinal complications is reported by Kim et al. [6]. Drug crystals from ion-exchange resins, such as sevelamer, polystyrene sulfonate, and cholestyramine, are shown to trigger NET formation and monocyte extracellular trap release and reduce metabolic activity and cell death in human intestinal epithelial cells in vitro, contributing to intestinal barrier dysfunction.

Because the emergence of the severe acute respiratory syndrome coronavirus 2 (SARS-CoV-2) coincided with this Special Issue and NETs were soon considered potential drivers of Coronavirus disease 2019 (COVID-19) [7], this Special Issue also contains two original research articles on NET formation and COVID-19. The first article addresses the correlation between immunoglobulin A2 (IgA2) antibodies against SARS-CoV-2 with NET formation and fatal outcomes [8]. IgA2 is able to activate immune cells and induces inflammation and NET formation. Therefore, Staats et al. investigated IgA2 responses against SARS-CoV-2 in plasma samples of 82 COVID-19 patients and compared antibody levels to C-reactive protein (CRP) and circulating extracellular DNA (ecDNA), which represent general inflammation and NET formation, respectively. Interestingly, the levels of SARS-CoV-2 immunoglobulin G antibodies were similar in all patient groups, whereas SARS-CoV-2 IgA2 antibodies were restricted to patients in the intensive care unit and correlated with CRP and ecDNA levels. In the second paper, Knopf et al. investigated changes in NET formation during the COVID-19 pandemic in a well-characterized Swedish cohort of patients with Systemic Lupus Erythematosus (SLE) [9]. COVID-19 and SLE are characterized, amongst others, by dysregulated type I interferon responses, an increased risk for thromboembolism, the robust activation of the complement system, and an imbalance in NET formation and clearance. The authors observed a change, especially in the activity of neutrophil elastase (NE) and the presence of NE-DNA complexes in the sera of patients taken during the pandemic vs. before the pandemic, independent of exposure to SARS-CoV-2.

Overall, this Special Issue highlights the role of NET formation not only in periodontal disease and salivary gland obstruction but also in the context of COVID-19. It shows how little is still known about the removal of NETs, demonstrating the need for further research not only on NET formation and its implication in health and disease but also on NET clearance.
